# The First Temporal and Spatial Assessment of *Vibrio* Diversity of the Surrounding Seawater of Coral Reefs in Ishigaki, Japan

**DOI:** 10.3389/fmicb.2016.01185

**Published:** 2016-08-08

**Authors:** A.K. M. R. Amin, Gao Feng, Nurhidayu Al-saari, Pedro M. Meirelles, Yohei Yamazaki, Sayaka Mino, Fabiano L. Thompson, Toko Sawabe, Tomoo Sawabe

**Affiliations:** ^1^Laboratory of Microbiology, Faculty of Fisheries Sciences, Hokkaido University, HakodateJapan; ^2^Institute of Biology, SAGE-COPPE, Federal University of Rio de Janeiro, Rio de JaneiroBrazil; ^3^Department of Nutrition, Hakodate Junior College, HakodateJapan

**Keywords:** vibrios, diversity dynamics, coral reef, seawater, environmental determinants

## Abstract

Coral reefs perform a major role in regulating marine biodiversity and serve as hotspot for highly dynamic and diverse microbiomes as holobionts. Corals around Ishigaki, however, are at risk due to tremendous stressors including elevation of seawater temperature, eutrophication and so on. However, no information is currently available on how *Vibrio* diversity fluctuates spatially and temporally due to environmental determinants in Ishigaki coral reef ecosystems. The aim of this study is to elucidate spatiotemporal *Vibrio* diversity dynamic at both community and population levels and to assess the environmental drivers correlated to *Vibrio* abundance and diversity. The *Vibrio* community identified based on *pyrH* gene phylogeny of 685 isolates from seawater directly connecting to Ishigaki coral holobionts consisted of 22 known and 12 potential novel *Vibrionaceae* species. The most prominent species were *V. hyugaensis*, *V. owensii* and *V. harveyi* followed by *V. maritimus/V. variabillis*, *V. campbellii*, *V. coralliilyticus*, and *Photobacterium rosenbergii*. The *Vibrio* community fluctuations, assessed by PCoA with UniFrac distance and clustering with Euclidiean distance were varied less not only by year but also by site. Interestingly, significant positive correlation was observed between rising seawater temperature and the abundance of *V. campbellii* (*r* = 0.62; *P* < 0.05) whereas the opposite was observed for *V. owensii* (*r* = -0.58; *P* < 0.05) and the C6 group of *V. hyugaensis* (*r* = -0.62; *P* < 0.05). AdaptML-based microhabitat differentiation revealed that *V. harveyi*, *V. campbellii*, *P. rosenbergii*, and *V. coralliilyticus* populations were less-ecologically distinctive whereas *V. astriarenae* and *V. ishigakensis* were ecologically diverse. This knowledge could be important clue for the future actions of coral conservation.

## Introduction

Coral reef ecosystem consists of a flexible consortium of eukaryotic and prokaryotic organisms as a holobiont and are rich in compounds for interacting each other as cross talks, protection of their territory and as triggers for these symbiotic association dynamics (Kooperman et al., 2007; Shnit-Orland and Kushmaro, 2009; Kvennefors et al., 2012). Coral hosts large, diverse and specific microbial populations, which have both important beneficial and harmful roles for the host (Ritchie and Smith, 2004; Rosenberg et al., 2007). More specifically, corals provide three habitats for bacteria (the surface mucus layer, coral tissue, and calcium carbonate skeleton) each of which harbors a distinct bacterial population (Koren and Rosenberg, 2006). Among the bacterial populations, vibrios have been recognized as important members of coral holobionts (Rohwer and Kelly, 2004; Vezzulli et al., 2010, 2012; Baker-Austin et al., 2013; Moreira et al., 2014; Rubio-Portillo et al., 2014; Munn, 2015). Some vibrios may establish mutualistic partnership with corals by releasing nutrients and secondary metabolites (Ritchie, 2006; Chimetto et al., 2008, 2009), while others play a major role in the disruption of coral health (Munn, 2015).

Coral and coral reefs are in steep decline worldwide due to the combined effects of various stressors such as global warming, pollution, overfishing and infectious diseases (Hughes et al., 2003; Weil et al., 2006; Dinsdale et al., 2008; Vezzulli et al., 2012; Baker-Austin et al., 2013). Rising seawater temperature related to global climate change is a big threat to coral health and is ultimately linked to increasing coral disease such as bleaching events (Tout et al., 2015). Impressive progress has been reported regarding characterization of the coral microbiota over the last decade with its altered composition often correlated to the appearance of signs and diseases and/or bleaching that ultimately suggest a link between microbes, global coral health and stability of reef ecosystems (Krediet et al., 2013). Among them, coral vibriosis is a well-known disease, of which occurrences are greatly influenced by increasing seawater temperature (Vezzulli et al., 2012; Baker-Austin et al., 2013; Munn, 2015). In particular, at warm temperatures, *V. shiloi* expresses a cell-surface adhesion protein that is required for bacterial attachment to the coral surface and simultaneously expresses Toxin-P that ultimately inhibits photosynthesis of the coral-endosymbiotic algae and super-oxide dismutase required for survival of this pathogen inside the coral (Rosenberg et al., 2007; Munn, 2015).

The coral reef ecosystem in Ishigaki Island in southwestern Japan is surrounded by well-developed fringing corals with a variety of mangroves and sandy or rocky shores (Sano, 2000; Shimomura and Naruse, 2015). Recently, these reefs have been severely stressed due to typhoons, coral bleaching and soil pollution. Red soil derived turbid seawater in Okinawa, Ishigaki, and Iriomote coral reef seawater adversely affects the photosynthesis of coral symbiont algae (Zooxanthelae) and also interfered with the settlement of coral larvae on the ocean bed which ultimately influenced coral damage (Kakuma and Kamimura, 2011). Due to this, eutrophication including inflow of red soil is an important threat to Ishigaki corals. More specifically, accumulation of red soil and high nutrient inputs due to land development since 1972, have severely affected the Ishigaki coral reef (Hasegawa, 2011). Rivers from the northern part of Ishigaki coral reef, originating from agricultural watershed may have detrimental effects on corals because exposure to silt and nutrient rich sediments may badly stress corals (Weber et al., 2006). In addition, run-off from soils after typhoons can easily damage coral as well (Harii et al., 2014). Presently, several reports are available regarding coral-microbiota from different parts of the world (Rohwer et al., 2002; Wild et al., 2004; Rosenberg et al., 2007; Chimetto et al., 2009; Alves et al., 2010; Krediet et al., 2013; Chimetto Tonon et al., 2015) but little is known about the Ishigaki coral reef microbial communities.

Therefore, the aims of this study are to characterize the *Vibrio* diversity spatially and temporally, to elucidate which environmental determinants [temperature or dissolved organic carbon (DOC)] correlate the *Vibrio* diversity dynamics and to characterize antimicrobial resistance profiles of potential coral pathogens.

## Materials and Methods

### Water Sampling and Isolation of Vibrios

**FIGURE 1:**
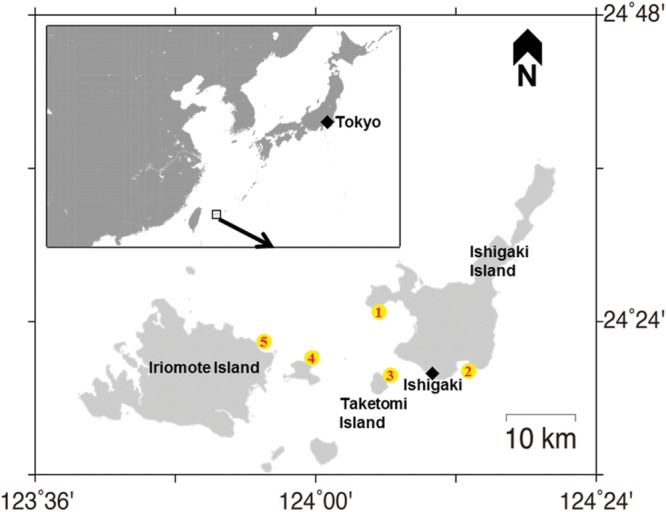
**Map showing the sampling locations.** Sites are marked with a yellow circle. (1) Osaki; (2) Miyara; (3) Taketomi; (4) Sekisei outer; (5) Iriomote in Ishigaki coral reef ecosystem.

Seawater samples were collected in 2012, 2013, and 2014 (June–July) from five sites (**Figure 1**) off the Ishigaki coral reef, Okinawa, Japan. The sampling sites were Miyara (24°20.5489′ N, 124°13.0408′ E), Osaki (24°25.4171′ N, 124°04.4956′ E), Taketomi (24°20.5260′ N, 124°05.6443′ E), Sekisei outer (24°21.7557′ N, 124°02.7190′ E), and Iriomote (24°23.208′ N, 123°55.681′ E) at depths of 3–4 m. More specifically, these seawater samples were within 2 m of coral holobionts and were collected by SCUBA diving and stored in clean containers and/or sterilized tubes. No specific permissions were required for the water sampling activities in these locations. The seawater samples were then brought back to the laboratory for bacterial isolation. A total of 0.2 mL of the seawater sample was directly spread on to thiosulfate-citrate-bile salt-sucrose (TCBS) agar plate (Nissui Pharmacy, Tokyo, Japan) and incubated at 25°C for 48 h. After incubation, numbers of individual colonies were counted manually to estimate ‘viable *Vibrio* counts.’ All colonies except those showing lack of growth were purified twice on the TCBS agar plates. The purity was checked using the ZoBell 2216E agar plate at 25°C. These strains were preserved in cryo-vials using the ZoBell broth supplemented with 20% (v/v) glycerol at -80°C.

### Molecular Phylogenetic Affiliation on the Basis of *pyrH* Gene Sequences to *Vibrionaceae* Species Groups

Molecular phylogenetic affiliation of all *Vibrio* isolates (*n* = 685) was performed on the basis of *pyrH* (uridylate kinase) gene sequences. A 550 bp of *pyrH* gene was amplified using pyrH80F and pyrH530R primers (Sawabe et al., 2007) as a colony suspension or a purified DNA as a PCR template. Genomic DNA was purified using a Promega Wizard Genomic DNA extraction system according to the protocol provided by the manufacturer. The PCR cycle consisted of an initial denaturation step at 95°C for 180 s and 30 cycles of an denaturation (95°C for 60 s), an annealing step (50°C for 60 s) and an extension step (72°C for 60 s). The PCR products were analyzed on 1.5% agarose gel and the PCR products producing a single band on agarose gels were purified using Promega Gel and PCR purification system (Promega, Madison, WI, USA). Approximately, 50 ng of template was directly sequenced using the pyrH80F or pyrH530R primer and a BigDye terminator sequencing kit version 3.1 (Life Technologies, Carlsbad, CA, USA) according to the protocol recommended by the manufacturer. DNA sequencing was performed with an Applied Biosystems model 373A automated sequencer. The same primers (pyrH80F or pyrH530R) were used for the sequencing.

Using our well-curated *pyrH* gene sequences, which could cover more than 145 known described species of *Vibrionaceae*, molecular phylogenetic affiliations of Ishigaki *Vibrio* isolates were performed. To retain the *pyrH* tree topology within *Vibrionaceae*, *pyrH* gene sequence of *Escherichia coli* W3110 (NC_010473) was included in the dataset as an out group. The sequences were aligned using Clustal X version 2.1 (Larkin et al., 2007) and the alignment was checked by eye and corrected manually. The position used for the affiliation corresponded to positions 171 to 543 in the *V. cholera* O1 Eltor N16961 (AE003852). *pyrH* gene phylogeny was reconstructed by neighbor-joining method (Saitou and Nei, 1987) with bootstrap values of 500 replicates implemented to MEGA programs version 6.06 (Tamura et al., 2013). Evolutionary distances were corrected using the Jukes-Cantor method. Clustering to a described species with more than 98.4% *pyrH* gene similarity was used for the final affiliation for species identification of Ishigaki coral reef isolates. For the affiliation of closed species in clusters harboring *V. harveyi*, *V. owensii*, *V. hyugaensis*, *V. communis*, *V. campbellii*, and *P. damselae* subsp. *piscicida*, a >99.7% cutoff was adopted to affiliate species. All *pyrH* and 16S rRNA genes sequences used in this study have been deposited in BaMBa under data package identification number pmeirelles.20.1. To further affiliate unassigned *pyrH* clusters to new species candidates, 16S rRNA gene sequences of 38 representative strains from *pyrH* unassigned clusters were obtained according to Al-saari et al. (2015). In brief, the amplification primers (24F and 1509R) used for PCR amplification gave a 1.5 kb long PCR product and corresponded to positions 25–1521 in the *E. coli* sequence. A 99.7% cut-off was used for the final affiliation of Ishigaki coral reef isolates to the new *Vibrionaceae* species candidate.

### Inferred Habitat Associations of *Vibrio* Community

To infer habitat associations of the *Vibrio* community in Ishigaki coral reef during a 3-years (2012–2014) assessment, PCoA based on unweighted UniFrac distance, hierarchical cluster analysis of *Vibrio* community composition and AdaptML was performed.

UniFrac distance was calculated using 658 *pyrH* gene sequences of *Vibrio* isolates and Quantitative Insights Into Microbial Ecology (QIIME) 1.9 software package (Caporaso et al., 2010) according to Yamazaki et al. (2016) except the use of *pyrH* gene phylogenetic tree. Relationships among *Vibrio* communities in accordance with sampling sites and years were visualized using PCoA. Hierarchical clustering analyses were performed with R Development Core Team (2011), except where indicated. Abundance and multivariate figures were plotted with packages ggplot2 and reshape (Wickham, 2007, 2009). The hierarchical cluster shown in **Figure 3B** was constructed using Ward’s minimum variance method (performed using “hclust” R function), which aims at finding compact, spherical clusters, on a Euclidian dissimilarity matrix (calculated using “dist” R function). *Vibrio* diversity indices (Shannon entropy and Shannon evenness [i.e., Hill’s Ratio]) and richness were calculated with the vegan R package (Oksanen et al., 2005).

The mathematical model (AdaptML), which uses a Hidden Markov Model, was used to predict the phylogenetic bounds of ecologically distinct *Vibrio* populations and their habitat composition (distribution among environmental categories). ‘Clusters of vibrios’ sequences were obtained using the software AdaptML as described previously (Hunt et al., 2008). In brief, the software combines genetic information embedded in sequence-based phylogenies and information about the ecology, herein the source and place of isolation, in order to identify genetically and ecologically distinct bacterial populations. AdaptML algorithms can account for environmental parameter discretization schemes and are based on the model concept of habitat (a place and related features that determines microbial distribution). Habitats are characterized by discrete probability distributions describing the likelihood that a strain adapted to a habitat will be sampled from a given ecological state, i.e., at a particular location in the water column). A maximum likelihood model is used for the evolution of habitat association on the tree (Hunt et al., 2008). The habitat-learning and clustering steps of AdaptML were performed using the default settings. Confident assignments are shown to fit ecological populations predicted by the model. The model threshold value was set at 0.05 and *E. coli* W3110 was used as an out-group. The bootstrap percentages analysis were rerun 100 times with the same phylogenetic tree to verify the stability of the predictions. The circular tree figure was drawn using online iTOL software (Letunic and Bork, 2007). To prevent numerical instabilities in AdaptML’s maximum likelihood computations, branches with zero length were assigned to the minimal observed non-zero branch length: 0.001. Clades supported in 80% of bootstraps are shown.

### Correlation between *Vibrionaceae* Species Groups and Environmental Determinants

Seawater environmental determinants including temperature and DOC were assessed. Water temperature was measured directly using a thermometer. DOC was determined using the method described by Grasshoff et al. (2009). Pearson correlation between each *Vibrio* abundance and environmental determinant was calculated using Microsoft Excel software.

## Results

### Diversity of Vibrios in Ishigaki Coral Reef Seawater

**FIGURE 2:**
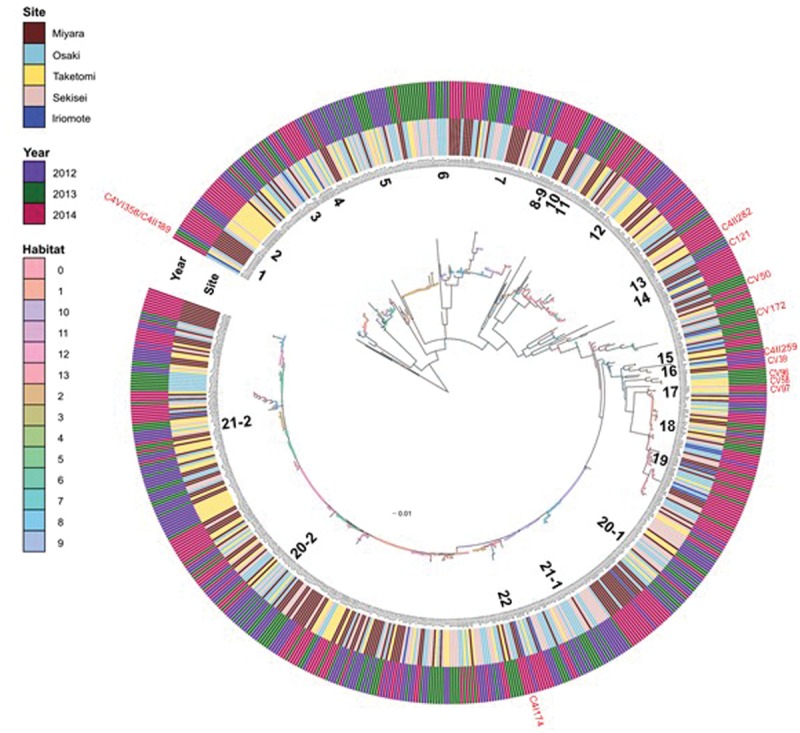
**Inferred habitats predicted by AdaptML model using *pyrH* gene sequences of *Vibrio* isolates from Ishigaki coral reef seawater.** Inner and outer ring indicate sampling sites and sampling years respectively. Predicted habitats are shown as nodes on the tree (habitat legend matches the colored circles). The closest named *Vibrio* species to numbered *Vibrio* populations’ are as follows- (1) *P. aphoticum*; (2) *V. astriarenae*; (3) *V. ishigakensis*; (4) *V. pelagius*; (5) *P. rosenbergii*; (6) *V. mediterranei*; (7) *V. variabillis/maritimus*; (8) *V. nigripulchritudo*; (9) *V. orientalis*; (10) *V. tubiashii*; (11) *V. alginolyticus*; (12) *V. coralliilyticus/neptunis*; (13) *V. ponticus*; (14) *V. azurius*; (15) *V. alfacsensis*; (16) *V. orientalis*; (17) *P. damsella* subsp. *piscicida*; (18) *V. harveyi*; (19) *V. campbellii*; (20-1) *V. owensii-*1; (20-2) *V. owensii*-2; (21-1) *V. hyugaensis*; (21-2) *V. hyugaensis*; (22) *V. communis*. Red marked strains, outside of the outer ring indicate the positions of putative new species candidates.

Our investigation of *Vibrio* diversity in Ishigaki coral reef seawater using *pyrH* gene phylogeny discovered a total of 44 clusters (Supplementary Table S1; **Figure 2**); 32 clusters were affiliated to 22 known *Vibrionaceae* species including 10 *V. hyugaensis* and 2 *P. damselae* subsp. *piscicida* subclusters, and the other 12 were affiliated to novel species candidates in *Vibrionaceae*. A total of 658 strains were identified as known *Vibrionaceae* species to *V. alfacsensis*, *V. alginolyticus*, *V. astriarenae*, *V. azureus*, *V. campbellii*, *V. communis*, *V. coralliilyticus/V. neptunis*, *V. harveyi*, *V. hyugaensis*, *V. ishigakensis*, *V. mediterranei*, *V. nigripulchritudo*, *V. orientalis*, *V. owensii*, *V. ponticus*, *V. pelagius*, *V. rotiferianus*, *V. tubiashii*, *V. variabillis/maritimus*, *Photobacterium aphoticum, P. damselae* subsp. *piscicida*, and *Photobacterium rosenbergii*.

However, 26 strains belonged to C121 (*n* = 5), CV39 (*n* = 2), CV50 (*n* = 4), CV58 (*n* = 1), CV96 (*n* = 1), CV97 (*n* = 1), CV172 (*n* = 1), C4I174 (*n* = 4), C4II189, C4II259 (*n* = 1), C4III282 (*n* = 3) and C4V358 clusters which were not affiliated to any known vibrios species (**Figure 2**; Supplementary Table S1). *V. hyugaensis* was the largest group of the *Vibrio* community in Ishigaki coral reef seawater (*n* = 200, 29.2%) and was mostly diversified into 10 different *pyrH* clusters (Supplementary Table S1). The occupation rates of each *V. hyugaensis* cluster were C71 (2.0%), C68 (4.5%), C6 (7.6%), C16 (1.9%), C49 (1.9%), C58 (3.7%), C46 (6.0%), C156 (0.1%), C164 (1.3%), and C4I100 (0.1%). *V*. *owensii*, a potent coral pathogen, was present in 21.6% of the total isolated vibrios. The third most abundant *Vibrio* group was *V. variabilis*/*maritimus* which was recorded in 8.8% of the isolated vibrios. Other prominent vibrios were *P. rosenbergii* (6.0%), *V. campbellii* (5.3%), and *V. harveyi* (4.5%). Some other recognized potential coral pathogens, *V. coralliilyticus* (*n* = 49, 7.2%) and *V. alginolyticus* (*n* = 5) were also found. Less significant amounts of identified vibrios (Supplementary Table S1) were *V. ponticus* (*n* = 3, 0.4%), *V. pelagius* (*n* = 9, 1.3%), *V. tubiashii* (*n* = 5, 0.7%), *V. rotiferianus* (*n* = 3, 0.4%), *P. aphoticum* (*n* = 3, 0.4%) and two recent newly described species, *V. astriarenae* (3.4%) and *V. ishigakensis* (4.2%). *V. nigripulchritudo* (*n* = 1), *V. alfacsensis* (*n* = 1), *V. orientalis* (*n* = 1), *V. azureus* (*n* = 1), and *V. mediterranei* (*n* = 1) were less highlighted vibrios.

### *Vibrio* Community Dynamics

**FIGURE 3:**
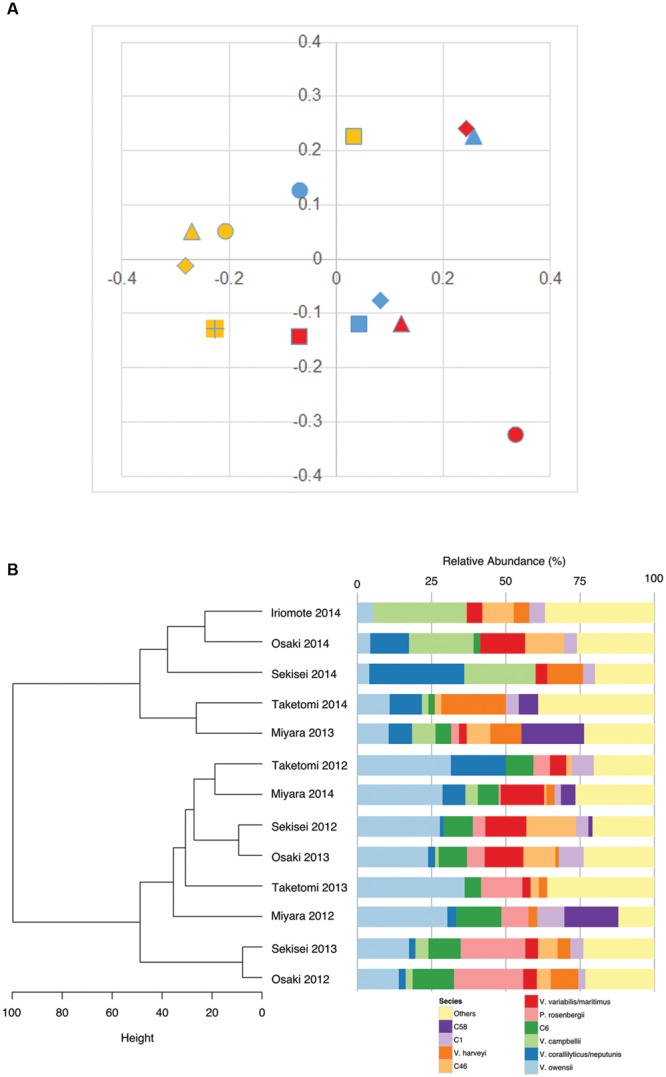
***Vibrio* community diversity dynamics in Ishigaki coral reef seawater. (A)** Multidimensional scaling plot (FastUnifrac:PCoA), isolates indicating colors and shapes: blue, 2012; red, 2013; yellow, 2014; dot, Taketomi; square, Miyara; diamond, Osaki; triangle, Sekisei; square (+), Iriomote; **(B)** dendrogram clustering of vibrios according to their relative abundances.

Unweighted FastUniFrac analysis revealed no-apparent grouping of *Vibrio* communities not only site by site but also year by year (**Figure 3A**). However, a simple cladogram constructed on the basis of relative abundances in the *Vibrio* community illustrated two apparent clusters in the *Vibrio* communities of Iriomote 2014, Osaki 2014, Sekisei 2014, Taketomi 2014 and Miyara 2013 and those of Taketomi 2012, Miyara 2014, Sekisei 2012, and Osaki 2013 (**Figure 3B**). Signatures of the former and the latter communities showed significant abundances (*P* < 0.05) of *V. campbellii*, *V. hyugaensis* (C6 group), and *V. owensii* respectively. Vibrios from Sekisei 2013 and Osaka 2012 were sub-clustered using a relative higher abundance of *P. rosenbergii*.

### Correlation between Environmental Variables and *Vibrio* Abundances

*Vibrio campbellii* abundance significantly increased (*r* = 0.62; *P <* 0.05) along with rising seawater temperature whereas *V. owensii* (*r* = -0.58; *P* < 0.05) and the C6 group of *V. hyugaensis* (*r* = -0.62; *P* < 0.05) abundances significantly decreased with an increase in seawater temperature (**Table 1**). Moreover, the correlation assessment between *Vibrio* abundances and DOC availability illustrated (**Table 1**) that *P. rosenbergii* and the *V. hyugaensis* (C6 group) increased along with increased DOC whereas *V. owensii*, *V. campbellii*, and *V. coralliilyticus* showed the opposite results.

**Table 1:** Correlation coefficient (*r*) and significant levels (*P*-values) obtained from the correlation analysis between major *Vibrio* abundances and environmental variables.

Vibrios	Environmental parameter	Correlation coefficient (*r*-value)	Significance (*P*-value)
*V. owensii*	Temperature	-0.58	0.038
*V. campbellii*	Temperature	0.62	0.023
*P. rosenbergii*	Temperature	-0.39	0.18
*V. coralliilyticus*	Temperature	0.16	0.60
*V. hyugaensis* (C6)	Temperature	-0.62	0.0013
*V. owensii*	DOC	-0.58	0.64
*V. campbellii*	DOC	-0.55	0.13
*P. rosenbergii*	DOC	0.60	0.086
*V. coralliilyticus*	DOC	0.25	0.52
*V. hyugaensis* (C6)	DOC	0.54	0.13

### Ecologically Distinct Populations among *Vibrio* Isolates

**FIGURE 4:**
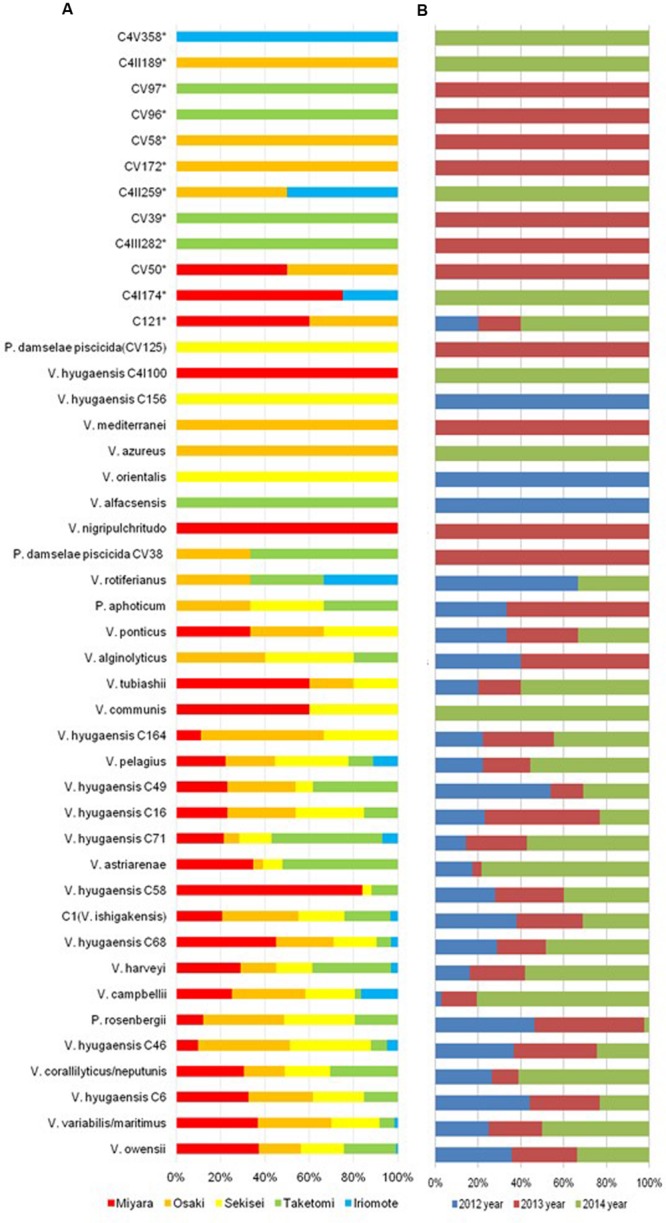
**Spatiotemporal *Vibrio* population dynamics in Ishigaki coral reef seawater. (A)** Spatial *Vibrio* population dynamics (red, brown, yellow, green, sky blue colors indicate sampling sites of Miyara, Osaki, Sekisei Outer, Taketomi, and Iriomote respectively); **(B)** Temporal *Vibrio* population dynamics (blue, violate, and green colors indicate sampling year of 2012, 2013, and 2014 accordingly).

The AdaptML analysis showed that the *Vibrio* isolates are distributed in eight habitat spectra (H-0, H-1, H-2, H-6, H-8, H-10, H-12, and H-13; **Figures 2** and **4**). Habitats are the part of an ecosystem (a spectrum of environment types) from where microbial populations are isolated. *V. owensii*, *V. harveyi*, *V. hyugaensis* (C71, C46, C6, C16, C49, C58, and C68 groups), *V. campbellii*, *V. ishigakensis*, *V. variabillis/V. maritimus*, *V. coralliilyticus/V. neptunis*, *P. rosenbergii*, and *V. pelagius* were observed from all the sites although *V. alfacsensis*, and *V. nigripulchritudo* were found only from Taketomi and Miyara respectively (**Figure 4**). Another two vibrios, *V. azureus* and *V. mediterranei* were specific for Osaki and one more single *Vibrio*, *V. orientalis* was recorded solely from Sekisei reef site. The spatiotemporal distribution of *V. campbelli* was likely to support the temperature dependent abundance from 2014 in these study areas (**Figure 4**). From all the predicted habitat spectra, H-10 and H-12 showed the strains which were isolated from all the sampling sites and sampling years with major isolates from Sekisei-2012 (41.7%) and Miyara-2014 (41.2%), respectively. Other habitats (H-0, H-1, H-2, H-6, H-8, H-13) showed the isolates (Supplementary Figure S1) from several sampling sites and years but not from all. The major isolates of H-0, H-1, H-2, H-6, H-8, and H-13 were obtained from Osaki-2014 (34.9%), Taketomi-2014 (34.9%), Osaki-2012 (20.2%), Osaki-2013 (33.7%), Miyara-2014 (78.6%), and Taketomi-2012 (55%) respectively.

The adapted predominant *Vibrio* species (Supplementary Table S2) in H-0 were *V. harveyi* (9.7%), *V. campbellii* (94.4%), *V. ishigakensis* (6.9%), *V. variabillis/V. maritimus* (11.7%), *V. coralliilyticus/V. neptunis* (32.7%), and *V. pelagius* (44.4%). The highlighted vibrios in H-1 were *V. harveyi* (80.6%) and *V. hyugaensis* C6 group (42.3%), *V. hyugaensis* C58 group (48.0%), *V. hyugaensis* C46 group (26.8%) and *V. astriarenae* (43.5%). H-2 comprised mostly of *V. owensii* (8.8%), *V. hyugaensis* C16 group (76.9%) and *P. rosenbergii* (97.6%). Two prevalent vibrios in H-6 were *V. owensii* (24.3%) and *V. ishigakensis* (44.8%). The most abundant vibrios in H-8 were *V. owensii* (16.2%) and *V. variabillis/V. maritimus* (28.3%). The dominant vibrios in H-10 were *V. owensii* (17.6%), *V. hyugaensis* C68 group (32.3%), *V. hyugaensis* C46 group (56.1%), *V. ishigakensis* (31.0%) and *V. variabillis/V. maritimus* (41.7%). In H-12 the preeminent vibrios were *V. owensii* (29.1%) and *V. hyugaensis*-C6 (30.8%). H-13 was found to contain *V. coralliilyticus–V. neptunis* (40.8%) and *V. astriarenae* (30.4%). Moreover, several potential novel vibrios (CV39, CV58, CV96, CV97) and one opportunistic coral pathogen *V. alginolyicus* (100%) were retrieved specifically from H-2.

AdaptML could also estimate the evolutionary history of ecological differentiation (Hunt et al., 2008). Interestingly, the recent ongoing adaptive radiations (with a shallow branch: meaning frequently changing adaptations) were observed as more likely to be in *V. astriarenae*, *V. ishigakensis*, *V. mediterranei*, *V. variabilis/V. maritimus*, *V. coralliilyticus/V. neputunis*, and *V. communis*/*V*. *owensii*/*V*. *hyugaensis*, however, no such radiations were observed in *V. campbellii*, *V. harveyi*, and *P. rosenbergii* (**Figure 2**).

## Discussion

Ishigaki coral reef ecosystems are known to be the largest and the most diverse reef ecosystems in Japan, but are in now extremely vulnerable. In this era of global warming, the surrounding seawater of coral reefs are one of the most important ecological niches as well as heat carriers. It is worth investigating the evolution of spatiotemporal *Vibrio* dynamics in oceanic fields not only for the conservation of fragile coral reefs but also for public health and aquaculture issues (Rubio-Portillo et al., 2014; Tout et al., 2015). We performed a 3 years survey to elucidate *Vibrio* diversity using well-curated *pyrH* gene sequence set to achieve fine scale discrimination (Thompson J.R. et al., 2005; Thompson et al., 2005a, 2007; Tall et al., 2013; Chimetto Tonon et al., 2015). The *pyrH* gene set allows us not only to affiliate *Vibrio* isolates to known described *Vibrionaceae* species or currently unknown new species candidates but also to elucidate the structure of regional populations of specific species and how they have diverged. Our study demonstrated that the *Vibrio* community of the coral surrounding seawater consisted of at least 22 known described species and 12 undescribed species candidates (26 isolates) which is likely to be a more diversified *Vibrio* community compared to those reported from tropical and temperate coral reef environments (Chimetto et al., 2008; Raina et al., 2009; Alves et al., 2010; Chimetto Tonon et al., 2015). Unfortunately, we did not assess the Ishigaki coral reef microbiota due to lack of coral samples, the seawater around Ishigaki coral reefs is likely to share major *Vibrionaceae* communities of reported microbiomes *V. communis*/*V. owensii*, *V. mediterranei* (*V. shiloi*), *V. harveyi*, *V. alginolyticus*, *V. campbellii*, *V. maritimus*/*V. variabilis*, *V. tubiashii*, *V. coralliilyticus*, *V. pelagius* consisting of coral holobionts (Alves et al., 2010; Chimetto Tonon et al., 2015). High abundance of *Harveyi* clade (*V. hyugaensis*, *V. communis, V. owensii, V. harveyi, V. campbellii*) and lower abundance of *Splendidus* clade species (*V. pelagius*) are the specific clues to help define subtropical Ishigaki coral reef microbiota (Hunt et al., 2008; Tall et al., 2013). *Coralliilyticus* clade species (*V. coralliilyticus, V. neptunis*), *Mediterranei* clade species (*V. mediterranei*, *V. maritimus, V. variabillis*) and *P. rosenbergii* are secondary significant members (>5% constitution) of the Ishigaki coral reef seawaters.

The availability of at least 12 novel species candidates (CV172, CV50, C4I174, CV96, CV58, CV39, CV97, C4II282, C121, C4II259, C4II189, and C4V358) in the Ishigaki coral reef ecosystem suggests important evidence of *Vibrionaceae* evolution. Relevant subsequent studies (Thompson et al., 2005b; Rosenberg et al., 2007; Reis et al., 2009; Moreira et al., 2014; Chimetto Tonon et al., 2015) reported that coral and surrounding seawater are prime sources in isolating novel vibrios and is an important habitat for studying the microevolution of vibrios. Comparing previously recorded vibrios from coral and reef ecosystems (Chimetto et al., 2009; Alves et al., 2010; Chimetto Tonon et al., 2015) we found mostly diversified populations including numerous vibrios unique to our studied reef locations. The retrieved unique *Vibrio* populations were *V. hyugaensis*, *V. ishigakensis*, *V. astriarenae*, *P. rosenbergii*, *P. damsella* subsp. *piscicida*, *P. aphoticum*, *V. nigripulchritudo*, *V. alfacsensis*, and *V. azureus* suggesting that Ishigaki coral reef locations are hotspots for versatile and dynamic *Vibrio* populations and may serve as diversified ecological niches.

This spatiotemporal survey also revealed that the community and population level dynamics in the surrounding seawater of coral holobionts in Ishigaki reef ecosystems can determine generalists and specialists (**Figures 2** and **4**). *V. owensii* and *V. hyugaensis* populations were considered to be the dominant generalist populations in the coral seawater ecosystems. The other prevalent generalists (Supplementary Table S1; **Figure 2**) in Ishigaki reef seawater were *V. variabillis*, *V. harveyi*, *V. campbellii*, *P. rosenbergii*, *V. coralliilyticus*, *V. astriarenae*, and *V. ishigakensis*.

Numerous previous reports suggested that vibrios from *Harveyi* clade appear to have ecologically diversified passively by invading new niches (Chimetto et al., 2008; Preheim et al., 2011; Chimetto Tonon et al., 2015). The observed diversities of *V. owensii* and *V. hyugaensis* populations were likely to be higher than those of *V. harveyi* and *V. campbellii* (**Figure 2**). *V. owensii* was isolated from cultured crustaceans in Australia as a member of *Harveyi* clade (Cano-Gomez et al., 2010). This species was also isolated from *Acropora* white syndrome lesions in American Samoa and is considered to be a putative coral pathogen (Ushijima et al., 2012; Wilson et al., 2012) and was also isolated from ‘*Montipora capitata*’ diseased-coral (Tissue loss disease: *Montipora* White Syndrome) in Hawaiian coral reefs. *V. hyugaensis* was isolated from a seawater sample collected in Miyazaki prefecture in Japan (Urbanczyk et al., 2015). It is a luminous *Vibrio* and several light-producing bacteria were reported from seawater samples taken from different sites in Miyazaki prefecture between 2010 and 2012 (Urbanczyk et al., 2014).

Specialist *Vibrio* populations in Ishigaki reef seawater showed site-specific distributions (Supplementary Table S1). *V. azureus* and *V. mediterranei* were isolated specifically from Osaki whereas, *V. nigripulchritudo*, *V. alfacsensis*, and *V. orientalis* were noted as region-specific in Miyara, Taketomi and Sekisei, respectively. Bacteria as coral holobionts may be host-specific or may display spatial variability which might show protective activities against pathogens or other detrimental agents by occupying entry niches and space (as competitors) or by production of antibiotic compounds (Rohwer et al., 2002; Mullen et al., 2004; Kelman et al., 2006; Ritchie, 2006). Moreover, the mathematical model AdaptML generated the environmental grouping (Supplementary Table S2) of the Ishigaki reef seawater *Vibrio* populations and detected both ecologically restricted and ecologically distinctive (Hunt et al., 2008) populations. Two proposed novel vibrios isolated from this location *V. astriarenae*, *Agarivorans* clade (Al-saari et al., 2015) and *V. ishigakensis*, *Halioticoli* clade (Gao et al., 2016) were found in more ecologically diverse populations (**Figure 2**). The former is agarolytic whereas the latter is non-motile and alginolytic. Benthic flora might determine the specific niches for these species.

One of the more intriguing findings disclosed by this study was the availability of versatile and dominant putative coral pathogenic vibrios, *V. owensii* and *V. coralliilyticus* (Supplementary Table S1; **Figure 2**) occupied 21.6 and 7.2%, respectively in our total analyzed vibrios. This may be due to higher global oceanic temperatures. Temperature influenced pathogenic potential of coral pathogenic vibrios may lead to an increase in incidences of diseases in marine environments. Swimming motility, swarming motility and protease activity have been shown to be related to the virulence of *V. owensii* (Ushijima et al., 2012). According to Kimes et al. (2012) for *V. coralliilyticus*, an increase in seawater temperature to more than 27°C plays a direct role in triggering virulence genes and other factors related to host degradation, secretion, antimicrobial resistance, and motility. The mechanism of virulence includes motility and chemotaxis that involve post-colonization with subsequent production of coral tissue damaging ‘zink-metalloproteases’ (Ben-Haim et al., 2003). Recently, Tout et al. (2015) demonstrated that raised-seawater temperature increase natural *Vibrio* abundances, particularly the notorious coral pathogen *V. coralliilyticus*, in coral ecosystems. However, our findings on *V. coralliilyticus* abundance were not significantly correlated to seawater temperature rises. Beyond the previously mentioned potential coral pathogens, we also identified *V. harveyi* (4.5%) and *V. alginolyticus* (0.7%) from the seawater. *V. alginolyticus* was found only in the single habitat H-2 suggesting that this bacterium may have a new ecological niche-partitioning in Ishigaki reef seawater. Other results associated with coral pathogenic vibrios (Luna et al., 2010; Zhenyu et al., 2013; Munn, 2015) show that most of them are opportunistic in nature and show their virulence under certain environmental conditions by overwhelming the host-defense, overgrowth and tissue destruction.

Environmental variables, particularly temperature are considered to be the prominent triggering factor in *Vibrio* ecology, population dynamics, physiological stress response and evolution (Sato et al., 2009). Elevated temperatures and high loads of nutrients particularly and DOC have direct impacts on coral holobiont microbial communities and ultimately make them diverse, abundant and even niche or host specific (Kline et al., 2006; Paz et al., 2007). Other findings similarly demonstrate that elevated nutrients (i.e., phosphate, nitrate, ammonia) and DOC in coastal waters have been a cause of reef decline (Bruno et al., 2003; Kline et al., 2006). In our assessment, only specific *Vibrionaceae* populations correlated with environmental parameters (e.g., temperature, DOC). *V. campbellii* abundance was significantly correlated to rises in seawater temperatures, *V. owensii* and C6 group of *V. hyugaensis* showed significant negative correlations with increasing seawater temperature. The optimum growth temperature of *V. hyugaensis* was reported to be 26°C and it could not grow at 37°C, which might reflect its low tolerance to high temperatures. These findings corroborated previous reports that thermal anomalies and nutrient-rich environment including DOC are well-known ecological triggers, involved in proliferation and increased abundance of holobiont microbial communities in coastal environments globally (Ducklow et al., 1986; Wild et al., 2004; Barott and Rohwer, 2012) along with reef ecosystems in southern Japan (Goto et al., 2010). Roder et al. (2014) also noted that coral associated bacterial communities including potential pathogens emerge and dominate during environmental stress and the shift of bacterial community may be a direct effect of temperature on growth of specific members of the microbial community (Garren et al., 2014).

## Conclusion

Our curated data set of *Vibrionaceae pyrH* gene sequences allows us to perform the first characterization of *Vibrio* diversity and assessment of spatiotemporal diversity dynamics of *Vibrio* populations both at community and population levels along with the relationship to environmental determinants from Ishigaki coral reef seawater. The determined *Vibrio* community of seawater directly connecting to Ishigaki coral holobionts included at least 22 known described species and 12 as yet undescribed species, which are more diversified compared to reported tropical and temperate reef vibrios. Several vibrios such as, *V. campbellii*, *V. owensii* and the C6 group of *V. hyugaensis* abundances seem to correlate with seawater temperature. *V. owensii* and C6 group of *V. hyugaensis* show significant negative correlations with increasing seawater temperature but *V. campbellii* is positively significant in this regard. This study also demonstrates the presence of most of the globally recognized opportunistic potential coral-pathogenic vibrios (*V. owensii, V. harveyi*, *V. alginolyticus*). This pioneering study on Ishigaki coral reef seawater vibrios both at community and population levels will be the important gateway for further deep research on vibrios in coral and reef systems.

## Author Contributions

Conceived and designed the experiments: AA, FT, and TomooS. Performed the experiments: AA, GF, NA, PM, YY, and SM. Analyzed the data: AA, GF, NA, PM, YY, and TomooS. Contributed reagents/materials/analysis tools: AA, GF, NA, PM, YY, SM, FT, TokoS, and TomooS. Wrote the paper: AA and TomooS. Critical review, ideas and suggestion given during manuscript preparation: GF, NA, PM, YY, SM, FT, and TokoS.

## Conflict of Interest Statement

The authors declare that the research was conducted in the absence of any commercial or financial relationships that could be construed as a potential conflict of interest.
