# Supplementary material for: The First Temporal and Spatial Assessment of Vibrio Diversity of the Surrounding Seawater of Coral Reefs in Ishigaki, Japan
Source: Front Microbiol. 2016 Aug 8;7:1185. doi: 10.3389/fmicb.2016.01185 (PMC4976104; doi:10.3389/fmicb.2016.01185)
Supplement: Supplementary file 1 [file Table_1.PDF]

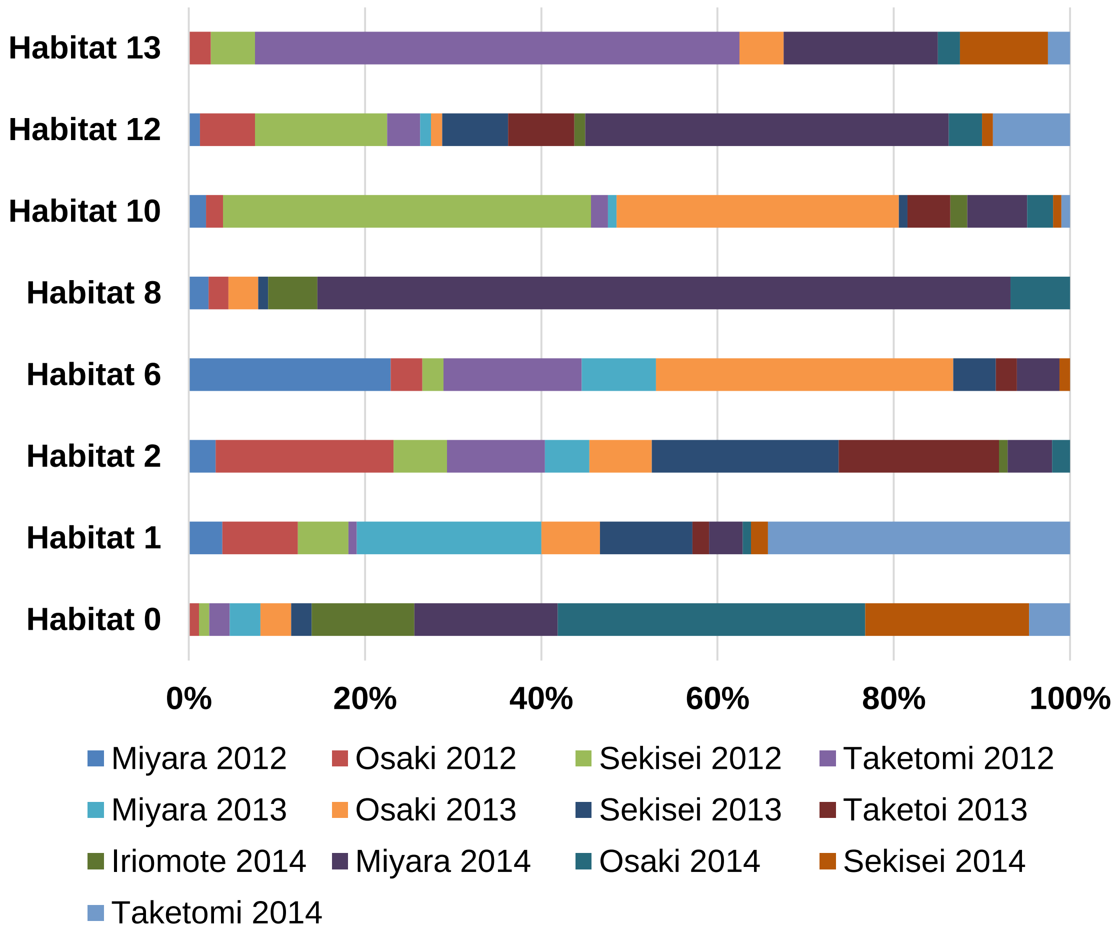

FIGURE S1 | Distribution of the environmental categories that compose AdaptML predicted each of the 8 habitats. Color legends indicate the *Vibrio* isolating different sampling sites and years.

Table S1| List of strains isolated from coral reef seawater at different sampling locations of Ishigaki Island.

**Species name**	Total no. of isolates (n, %)	**Sampling locations**				
		Miyara	Osaki	Taketomi	Sekisei	Iriomote
V. owensii	148 (21.6)	55	28	35	29	1
V. variabillis/maritimus	60 (8.8)	22	20	4	13	1
V. hyugaensis (C6)	52 (7.6)	17	15	8	12	0
V. coralliilyticus/neptunis	49 (7.2)	15	9	15	10	0
V. hyugaensis (C46)	41 (6.0)	4	17	3	15	2
P. rosenbergii	41 (6.0)	5	15	8	13	0
V. campbellii	36 (5.3)	9	12	1	8	6
V. harveyi	31(4.5)	9	5	11	5	1
V. hyugaensis (C68)	31 (4.5)	14	8	2	6	1
V. ishigakensis (C1)	29 (4.2)	6	10	6	6	1
V. hyugaensis (C58)	25 (3.7)	21	0	3	1	0
V. astriarenae (C7)	23 (3.4)	8	1	12	2	0
V. hyugaensis (C71)	14 (2.0)	3	1	7	2	1
V. hyugaensis (C16)	13 (1.9)	3	4	2	4	0
V. hyugaensis (C49)	13 (1.9)	3	4	5	1	0
V. pelagius	9 (1.3)	2	2	1	3	1
*V. hyugaensis (*C164)	9 (1.3)	1	5	0	3	0
V. communis	5 (0.7)	3	0	0	2	0
V. tubiashii	5 (0.7)	3	1	0	1	0
V. alginolyticus	5 (0.7)	0	2	1	2	0
V. ponticus	3 (0.4)	1	1	0	1	0
P. aphoticum	3 (0.4)	0	1	1	1	0
V. rotiferianus	3 (0.4)	0	1	1	0	1
P. damsellae (CV38)	3 (0.4)	0	1	2	0	0
V. nigripulchritudo	1 (0.1)	1	0	0	0	0
V. alfacsensis	1 (0.1)	0	0	1	0	0
V. orientalis	1 (0.1)	0	0	0	1	0
V. azureus	1 (0.1)	0	1	0	0	0
V. mediterranei	1 (0.1)	0	1	0	0	0
V. hyugaensis (C156)	1 (0.1)	0	0	0	1	0
V. hyugaensis (C4I100)	1 (0.1)	1	0	0	0	0
P. damsellae (CV125)	1 (0.1)	0	0	0	1	0
C121*	5 (0.6)	3	2	0	0	0
C4I174*	4 (0.6)	3	0	0	0	1
CV50*	4 (0.6)	2	2	0	0	0
C4III282*	3 (0.4)	0	0	3	0	0
CV39*	2 (0.3)	0	0	2	0	0
C4II259*	2 (0.3)	0	1	0	0	1
CV172*	1 (0.1)	0	1	0	0	0
CV58*	1 (0.2)	0	1	0	0	0
CV96*	1 (0.1)	0	0	1	0	0
CV97*	1 (0.1)	0	0	1	0	0
C4II189*	1 (0.1)	0	1	0	0	0
C4V358*	1 (0.1)	0	0	0	0	1

*** new species candidate.**

Table S2| Distribution (number) of vibrios that compose each of the 8 habitats inferred by AdaptML

**Vibrios**	**H-0**	**H-1**	**H-2**	**H-6**	**H-8**	**H-10**	**H-12**	**H-13**
V. owensii	1	0	13	36	24	26	43	5
V. variabillis/maritimus	7	1	6	0	17	25	0	4
V. hyugaensis (C6)	3	22	0	4	2	3	16	2
V. coralliilyticus/neptunis	16	5	0	4	4	0	0	20
V. hyugaensis (C46)	2	11	0	0	1	23	3	1
P. rosenbergii	0	0	40	1	0	0	0	0
V. campbellii	34	1	0	0	0	0	1	0
V. harveyi	3	25	0	0	3	0	0	0
V. hyugaensis (C68)	2	5	0	5	9	10	0	0
V. ishigakensis (C1)	2	2	3	12	0	10	0	0
V. hyugaensis (C58)	0	2	12	1	7	1	0	2
V. astriarenae (C7)	0	9	0	0	7	0	0	7
V. hyugaensis (C71)	2	4	0	3	1	0	4	0
V. hyugaensis (C16)	0	2	10	0	1	0	0	0
V. hyugaensis (C49)	3	0	0	4	0	0	5	1
V. pelagius	4	0	4	0	1	0	0	0
*V. hyugaensis (*C164)	0	1	0	1	2	2	3	0
V. communis	1	0	0	0	3	1	0	0
V. tubiashii	0	0	2	0	3	0	0	0
V. alginolyticus	0	0	5	0	0	0	0	0
V. ponticus	0	0	0	0	0	3	0	0
P. aphoticum	0	0	3	0	0	0	0	0
V. rotiferianus	0	0	3	0	0	0	0	0
P. damsellae (CV38)	0	0	3	0	0	0	0	0
V. nigripulchritudo	0	1	0	0	0	0	0	0
V. alfacsensis	0	0	0	0	0	0	0	1
V. orientalis	0	0	0	0	0	1	0	0
V. azureus	1	0	0	0	0	0	0	0
V. mediterranei	0	0	0	1	0	0	0	0
V. hyugaensis (C156)	0	0	0	0	0	0	1	0
V. hyugaensis (C4I100)	0	0	0	0	0	1	0	0
P. damselae (CV125)	0	0	0	1	0	0	0	0
C121*	0	0	0	0	5	0	0	0
C4I174*	0	0	0	0	4	0	0	0
CV50*	0	0	0	4	0	0	0	0
C4III282*	0	3	0	0	0	0	0	0
CV39*	0	0	2	0	0	0	0	0
C4II259*	1	0	0	0	1	0	0	0
CV172*	0	0	0	1	0	0	0	0
CV58*	0	0	1	0	0	0	0	0
CV96*	0	0	1	0	0	0	0	0
CV97*	0	0	1	0	0	0	0	0
C4II189*	1	0	0	0	0	0	0	0
C4V358*	1	0	0	0	0	0	0	0

*** new species candidate.**
